# Trends in Brazilian market of antidepressants: A five-year dataset analysis

**DOI:** 10.3389/fphar.2022.893891

**Published:** 2022-10-04

**Authors:** Rogério Hoefler, Taís Freire Galvão, Inês Ribeiro-Vaz, Marcus Tolentino Silva

**Affiliations:** ^1^ Department of Community Medicine, Health Information and Decision, Faculty of Medicine, University of Porto, Porto, Portugal; ^2^ Federal Council of Pharmacy, Brasília, Brazil; ^3^ Faculty of Pharmaceutical Sciences, University of Campinas, Campinas, Brazil; ^4^ Porto Pharmacovigilance Centre, Faculty of Medicine, University of Porto, Porto, Portugal; ^5^ Center for Health Technology and Services Research (CINTESIS), Faculty of Medicine, University of Porto, Porto, Portugal; ^6^ University of Sorocaba, Sorocaba, Brazil

**Keywords:** antidepressive agents, drug utilization, market research, commerce, Brazil

## Abstract

**Introduction:** Depression is an emotional disorder associated with morbidities and disabilities worldwide. The growing use of antidepressants is a concern for health managers because there are still unanswered questions on the effectiveness and safety of these medicines. Drug sales have increased in Brazil in recent years, but investigations on antidepressants sales are not available. We aimed to describe the trends in the antidepressant commerce in Brazil in a five-year period.

**Materials and Methods:** We performed an ecological study on antidepressant sales in Brazil, from November 2014 to October 2019, using data from IQVIA™, a data provider of pharmaceutical sales. Antidepressants were coded by the Anatomical Therapeutic Chemical classification system, and sales were presented in defined daily doses (DDDs) and DDDs per 1,000 inhabitants per day (DIDs). The results were expressed in absolute quantities and growth rates.

**Results:** The analyzed dataset contained 23 active substances in 780 products. The total sales of antidepressants increased from 23.3 DIDs in November 2014 to 38.3 DIDs in October 2019 (*p* = 0.002). Selective serotonin reuptake inhibitors were the most sold category of drugs (+5.7 million DDDs) in the period. ‘Other’ antidepressants presented the largest growth rate (104.7%). Individually, the most sold active substance was escitalopram (+1.8 million DDDs), and vortioxetine had the largest growth rate (336.2%). Tricyclic sales remained unchanged, and monoamine oxidase inhibitors had low and even decreasing sales (−9.5%).

**Discussion:** The total sales of antidepressants increased in Brazil from November 2014 to October 2019. The higher sale volumes of selective serotonin reuptake inhibitors and higher growth rate of ‘other’ antidepressants, with low sale volume of tricyclics and a decrease of monoamine oxidase inhibitors, suggest the replacement of older drugs by newer ones following a global trend. Therapeutic advances and commercial promotion efforts on new products might explain these findings.

## Introduction

Depression is an important cause of morbidity and disability worldwide (GBD 2019 Diseases and Injuries Collaborators, 2020). In 2019, it was estimated to affect 280 million people, accounting for the second-leading cause of disability globally (GBD 2019 Diseases and Injuries Collaborators, 2020). In Brazil, an emerging economy with high social disparities, depressive disorders affected 4.5 million people in 1990 and 7.2 million in 2017 (Bonadiman et al., 2021). In 2019, according to the Brazilian National Health Survey, 10.2% of adults reported having received a previous diagnosis of depression, and 48% of them had been using antidepressant drugs (Instituto Brasileiro de Geografia e Estatística, 2020a).

A growth in antidepressant use is consistent across different settings worldwide (Domínguez et al., 2017; Soleymani et al., 2018; Bliźniewska-Kowalska et al., 2020; Devos et al., 2020; Luo et al., 2020; Yu et al., 2020). Trends of antidepressant commerce seem to be associated to sociodemographic, cultural, and economic determinants (Loyola Filho et al., 2014; Gomez-Lumbreras et al., 2019). In Europe, the prescribing pattern was strongly influenced by previous use of antidepressants; physician (age, gender, and medical specialty) and patient factors—severity of depression, age, education, smoking; and current physical conditions (Bauer et al., 2008). Reimbursement policies (Forns et al., 2019), marketing efforts, regulations, adverse effect spectrum, and patient preferences may also influence the patterns of antidepressant use (Luo et al., 2021).

Aggregated information on drug sales may help researchers to understand pharmaceutical market trends and allow comparing different regions into a country or different countries. The World Health Organization (WHO) recommends the Anatomic Therapeutic Chemical (ATC) and the Defined Daily Dose (DDD) methodology, developed by the Norwegian Institute of Public Health, as the gold standard for drug utilization monitoring and research (Hollingworth and Kairuz, 2021). The DDD is assumed as the average maintenance daily dose for a medicine used on its main indication in adults. It is a global standardized metric that provides a fixed unit of measurement independent of price, currencies, and package size and strength and allows trends in drug consumption to be assessed and comparisons between countries and population groups to be performed. Sales or prescription data presented in DDD in relation to the population size may provide a rough estimate of the proportion of the study population treated daily with a particular drug or group of drugs (Hollingworth and Kairuz, 2021).

Medicalization of life with psychotropic drugs is a recurrent and complex circumstance due to its assignable central role in transforming human conflict and psychological suffering in solvable problems (Rodrigues et al., 2020). Drug utilization studies in this field may be an important strategy to assess this phenomenon.

Despite the high burden of depressive disorders and feasibility of performing drug utilization studies based on administrative or health services databases, for example, Brazil lacks assessments of antidepressant sales. Pooled data from the pharmaceutical market, including sale registries from drug companies, wholesalers, and retailers, could be useful for estimating the total quantities and trends of antidepressant use in this population. Data from companies specialized in monitoring of the pharmaceutical market, as IQVIA, former IMS Health, may be a source to investigate tendencies of drug sales, in this sense (Hollingworth and Kairuz, 2021).

The aim of this study is to describe the trends of antidepressant sales in Brazil in a five-year period and assess main fluctuations in sales of drugs and pharmacological categories.

## Materials and methods

### Study design

It is an exploratory ecological study based on the analysis of data of antidepressant sales in Brazil from November 2014 to October 2019.

### Setting

The data of antidepressant sales in Brazil were provided by IQVIA™, a company that collects daily information on the local pharmaceutical market. The information is based on private pharmacies, hospitals, governments, their wholesaler providers, and direct sales from drug companies. The different sources of sales are complementary and used for cross-checking to avoid double counting. The initial dataset provided data on product name, drug company name, active substance name, European Pharmaceutical Market Research Association (EphMRA) classification code, pharmaceutical form, dosage per package, and quantity of packages sold. The data provided by IQVIA™ contained all N6-coded drugs (psychoanaleptics excluding anti-obesity preparations), which include N6A—antidepressants and mood stabilizers; N6B—psychostimulants; N6C—psycholeptic-psychoanaleptic combinations; N6D—nootropics; N6E—neurotonics; and other miscellaneous products (Norwegian Institute of Public Health, 2020). Only drugs coded as N6A were initially included for analysis. We checked the regulatory status of each included product that presented no one sold unity in at least one studied period through two databases: Brazilian Health Regulatory Agency—Anvisa, public access on: https://consultas.anvisa.gov.br/#/medicamentos/; and Health Environment Legal Prevention & Safety—by Optionline, paid subscription on: https://i-helps.optionline.com/.

We selected all antidepressants in monotherapy presentation. Products with expired market license before the analyzed period were not excluded if there were registries of sales at least in the first studied period. Combination products that included antidepressants in their preparation and mood stabilizers and herbal drugs were excluded. Antidepressants were identified by international nonproprietary name and were recoded according to the ATC classification system. The ATC codes and DDD value data were obtained in March 2020 from the WHO Collaborating Centre for Drug Statistics Methodology website: https://www.whocc.no/atc_ddd_index/.

### Variables

The primary outcome was the DDD per 1,000 inhabitants per day (DID) (Hollingworth and Kairuz, 2021) and the growth rate between 12-month periods and for the total five-year period.

To calculate the DID values, we converted the yearly sold package unities of each antidepressant drug to DDD unities. First, the number of DDDs per package was calculated by dividing the total dosage of the active substance per package (in milligrams) by the corresponding DDD value for the substance; then, the result was multiplied by the number of packages of that product sold in each 12-month period of this study (2014–5: November 2014 to October 2015; 2015–6: November 2015 to October 2016; 2016–7: November 2016 to October 2017; 2017–8: November 2017 to October 2018; and 2018–9: November 2018 to October 2019). The quantities of products, in DDDs per 12-month period, were pooled by the active substance and by the pharmacological category. To calculate the DID, we considered the Brazilian adult population (≥18 years old) as estimated by the Brazilian Institute of Geography and Statistics for each corresponding year, considering 2015 population for the 2014–5 period and so on (Instituto Brasileiro de Geografia e Estatística, 2020b).

The growth rate was assumed as the percent of change on the quantity of antidepressants sold per substance and per pharmacological category, calculated each 12-month period against the previous 12-month period and the fifth period (2018–9) against the first period (2014–5).

The drugs were analyzed both individually and into their respective ATC pharmacological categories: tricyclic antidepressants, named as “non-selective monoamine reuptake inhibitors” at the ATC classification (N06AA); selective serotonin reuptake inhibitors (N06AB); monoamine oxidase inhibitors (N06AF and N06AG); and ‘other’ antidepressants (N06AX), including serotonin–norepinephrine reuptake inhibitors.

### Statistical methods

We described the frequency of sales in DDD and DID and the growth rate in each period. The Chi2 trend value was calculated for assessing whether there was a significant change of DID data between 2014–5 and 2018–9 periods by the active substance, by the pharmacological category, and by the total number of antidepressants sold. Excel Microsoft Office 365 was used for plotting and analyzing the data and for creating tables and figures.

## Results

**FIGURE 1:**
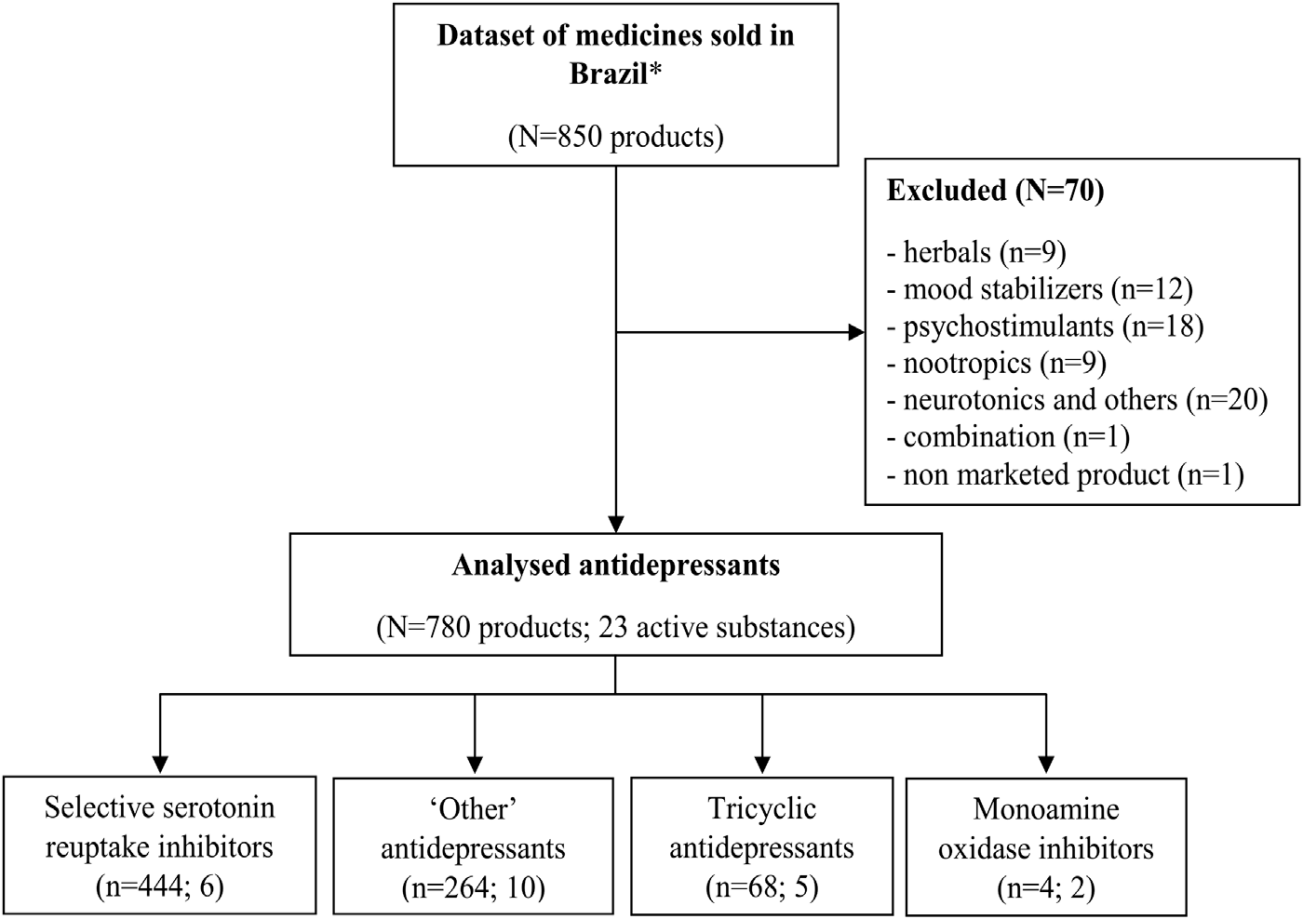
Antidepressant selection for analysis. Note: *N6 coded by EphMRA - psychoanaleptics excluding antiobesity preparations.

The initial dataset contained 850 drug products coded as N6 according to the EphMRA classification system. Seventy of them were excluded from the analysis, out of which 59 were not antidepressants, nine were herbals (*Hypericum perforatum*), one was a combination (amitriptyline + carbamazepine), and one product based on milnacipran substance was not marketed anymore; the production of products based on moclobemide and reboxetine was discontinued before the analyzed period, but there were sales registered until the third studied period (2016–7). The final dataset for analysis contained 23 active substances in 780 products (Figure 1).

The total sales of antidepressants in Brazil increased from 23.3 DIDs in 2014–5 to 38.3 DIDs in 2018–9 (*p* = 0.002) (Table 1). Selective serotonin reuptake inhibitors were the main antidepressant category sold in total in the five-year period (+5.7 million DDDs) (Table 1), and “other” antidepressants represented the largest growth rate (104.7%) (Table 2).

**TABLE 1:** Sales of antidepressants in Brazil in defined daily doses and DDDs per 1,000 inhabitants per day (DID), from November 2014 to October 2019.

	2014–5		2015–6		2016–7		2017–8		2018–9		2014–5 to 2018–9^a^
	DDDs	DID	DDDs	DID	DDDs	DID	DDDs	DID	DDDs	DID	*p* Value
Tricyclics	78,619,010	1.5	84,355,418	1.6	99,209,475	1.8	107,482,893	1.9	106,708,436	1.9	0.735
Clomipramine (N06AA04)	10,916,087	0.2	11,363,251	0.2	11,667,746	0.2	11,457,774	0.2	10,967,892	0.2	0.660
Imipramine (N06AA02)	6,748,429	0.1	5,684,769	0.1	6,632,455	0.1	7,080,235	0.1	6,642,915	0.1	0.732
Maprotiline (N06AA21)	673,775	<0.1	562,685	<0.1	559,105	<0.1	516,075	<0.1	148,560	<0.1	0.959
Nortriptyline (N06AA10)	17,858,048	0.3	18,443,013	0.3	19,857,744	0.4	20,199,626	0.4	20,935,461	0.4	0.544
Amitriptyline (N06AA09)	42,422,671	0.8	48,301,700	0.9	60,492,425	1.1	68,229,183	1.2	68,013,608	1.2	0.274
Serotonin reuptake inhibitors	882,376,221	16.5	991,672,616	18.2	1,124,176,242	20.4	1,263,422,727	22.6	1,441,425,991	25.4	0.028
Citalopram (N06AB04)	142,388,747	2.7	146,119,800	2.7	149,238,298	2.7	152,508,960	2.7	157,719,737	2.8	0.942
Escitalopram (N06AB10)	235,642,230	4.4	289,991,634	5.3	345,463,452	6.3	419,969,042	7.5	510,029,115	9.0	0.029
Fluoxetine (N06AB03)	176,235,494	3.3	193,339,903	3.6	230,445,202	4.2	242,224,949	4.3	248,871,359	4.4	0.548
Fluvoxamine (N06AB08)	7,176,518	0.1	7,927,343	0.1	9,025,928	0.2	11,311,260	0.2	14,617,223	0.3	0.736
Paroxetine (N06AB05)	114,685,255	2.1	121,758,830	2.2	129,780,535	2.4	142,823,123	2.6	153,259,460	2.7	0.704
Sertraline (N06AB06)	206,247,977	3.9	232,535,106	4.3	260,222,827	4.7	294,585,393	5.3	356,929,097	6.3	0.215
Monoamine oxidase inhibitors	969,890	<0.1	886,280	<0.1	870,010	<0.1	920,740	<0.1	931,260	<0.1	0.990
Tranylcypromine (N06AF04)	887,480	<0.1	859,160	<0.1	870,000	<0.1	920,740	<0.1	931,260	<0.1	0.999
Moclobemide (N06AG02)^b^	82,410	<0.1	27,120	<0.1	10	<0.1	-	-	-	-	0.969
‘Other’ antidepressants	288,136,417	5.4	336,584,777	6.2	390,931,060	7.1	496,055,706	8.9	625,533,858	11.0	0.015
Agomelatine (N06AX22)	6,219,948	0.1	5,486,866	0.1	5,232,976	0.1	5,374,642	0.1	5,909,652	0.1	0.972
Bupropion (N06AX12)	48,285,933	0.9	50,703,057	0.9	47,809,732	0.9	55,283,790	1.0	63,375,420	1.1	0.822
Desvenlafaxine (N06AX23)	40,625,704	0.8	47,090,498	0.9	57,131,472	1.0	102,762,003	1.8	165,977,309	2.9	0.013
Duloxetine (N06AX21)	54,748,476	1.0	69,504,263	1.3	87,037,805	1.6	108,239,693	1.9	128,566,076	2.3	0.220
Mirtazapine (N06AX11)	27,523,836	0.5	32,498,303	0.6	36,874,338	0.7	42,929,556	0.8	52,202,360	0.9	0.572
Reboxetine (N06AX18)^b^	57,180	<0.1	45,460	<0.1	1,250	<0.1	-	-	-	-	0.974
Tianeptine (N06AX14)	201,050	<0.1	181,250	<0.1	168,420	<0.1	154,610	<0.1	151,330	<0.1	0.986
Trazodone (N06AX05)	18,122,893	0.3	20,783,240	0.4	24,834,202	0.4	29,680,893	0.5	34,715,268	0.5	0.639
Venlafaxine (N06AX16)	92,351,397	1.7	106,960,800	2.0	123,166,995	2.2	139,723,755	2.5	159,457,978	2.1	0.410
Vortioxetine (N06AX26)^c^	0	0.0	3,331,040	0.1	8,673,870	0.2	11,906,750	0.2	15,178,465	0.3	0.405
Total	1,250,101,538	23.3	1,413,499,091	26.0	1,615,186,787	29.3	1,867,882,066	33.4	2,174,599,545	38.3	0.002

Notes:

a 12-month periods: 2014–5: November 2014 to October 2015; 2015–6: November 2015 to October 2016; 2016–7: November 2016 to October 2017; 2017–8: November 2017 to October 2018; 2018–9: November 2018 to October 2019.

b Products based on reboxetine and moclobemide were discontinued in 2018 and 2019, respectively, there were sales registered until the third studied period (2016–7).

c Product based on vortioxetine was introduced in 2015.

**TABLE 2:** Sale growth rate of antidepressants in Brazil, from November 2014 to October 2019.

	Sale growth rate between periods (%)				
	2014–5 to 2015–6	2015–6 to 2016–7	2016–7 to 2017–8	2017–8 to 2018–9	2014–5 to 2018–9^a^
Tricyclics	5.7	15.9	6.8	−2.1	28.0
Clomipramine (N06AA04)	2.5	1.2	−3.2	−5.6	−5.3
Imipramine (N06AA02)	−17.0	14.9	5.2	−7.5	−7.2
Maprotiline (N06AA21)	−17.7	−2.1	−9.0	−71.6	−79.2
Nortriptyline (N06AA10)	1.7	6.1	0.3	2.2	10.5
Amitriptyline (N06AA09)	12.1	23.4	11.2	−1.7	51.2
Serotonin reuptake inhibitors	10.7	11.7	10.8	12.5	54.0
Citalopram (N06AB04)	1.1	0.6	0.7	1.9	4.4
Escitalopram (N06AB10)	21.2	17.4	19.8	19.7	104.1
Fluoxetine (N06AB03)	8.1	17.4	3.6	1.3	33.2
Fluvoxamine (N06AB08)	8.8	12.2	23.5	27.4	92.1
Paroxetine (N06AB05)	4.6	5.0	8.5	5.8	26.0
Sertraline (N06AB06)	11.0	10.3	11.6	19.4	63.2
Monoamine oxidase inhibitors	−10.0	−3.3	4.3	−0.3	−9.5
Tranylcypromine (N06AF04)	−4.7	−0.2	4.3	−0.3	−1.1
Moclobemide (N06AG02)^b^	−67.6	−100.0	−100.0	0.0	−100.0
‘Other’ antidepressants	15.1	14.4	25.1	24.3	104.7
Agomelatine (N06AX22)	−13.1	−6.0	1.2	8.4	−10.4
Bupropion (N06AX12)	3.4	−7.1	14.0	13.0	23.8
Desvenlafaxine (N06AX23)	14.2	19.5	77.3	59.2	285.2
Duloxetine (N06AX21)	25.0	23.4	22.6	17.1	121.4
Mirtazapine (N06AX11)	16.3	11.8	14.8	19.9	78.8
Reboxetine (N06AX18)^b^	−21.7	−97.3	−100.0	0.0	−100.0
Tianeptine (N06AX14)	−11.2	−8.4	−9.5	−3.5	−29.0
Trazodone (N06AX05)	12.9	17.7	17.8	15.3	80.6
Venlafaxine (N06AX16)	14.1	13.5	11.8	12.5	62.8
Vortioxetine (N06AX26)^c^	-	156.6	35.3	25.7	336.2

Notes:

a 12-month periods: 2014–5: November 2014 to October 2015; 2015–6: November 2015 to October 2016; 2016–7: November 2016 to October 2017; 2017–8: November 2017 to October 2018; 2018–9: November 2018 to October 2019.

b Products based on reboxetine and moclobemide were discontinued in 2018 and 2019, respectively, there were sales registered until the third studied period (2016–7).

c Product based on vortioxetine was introduced in 2015.

**FIGURE 2:**
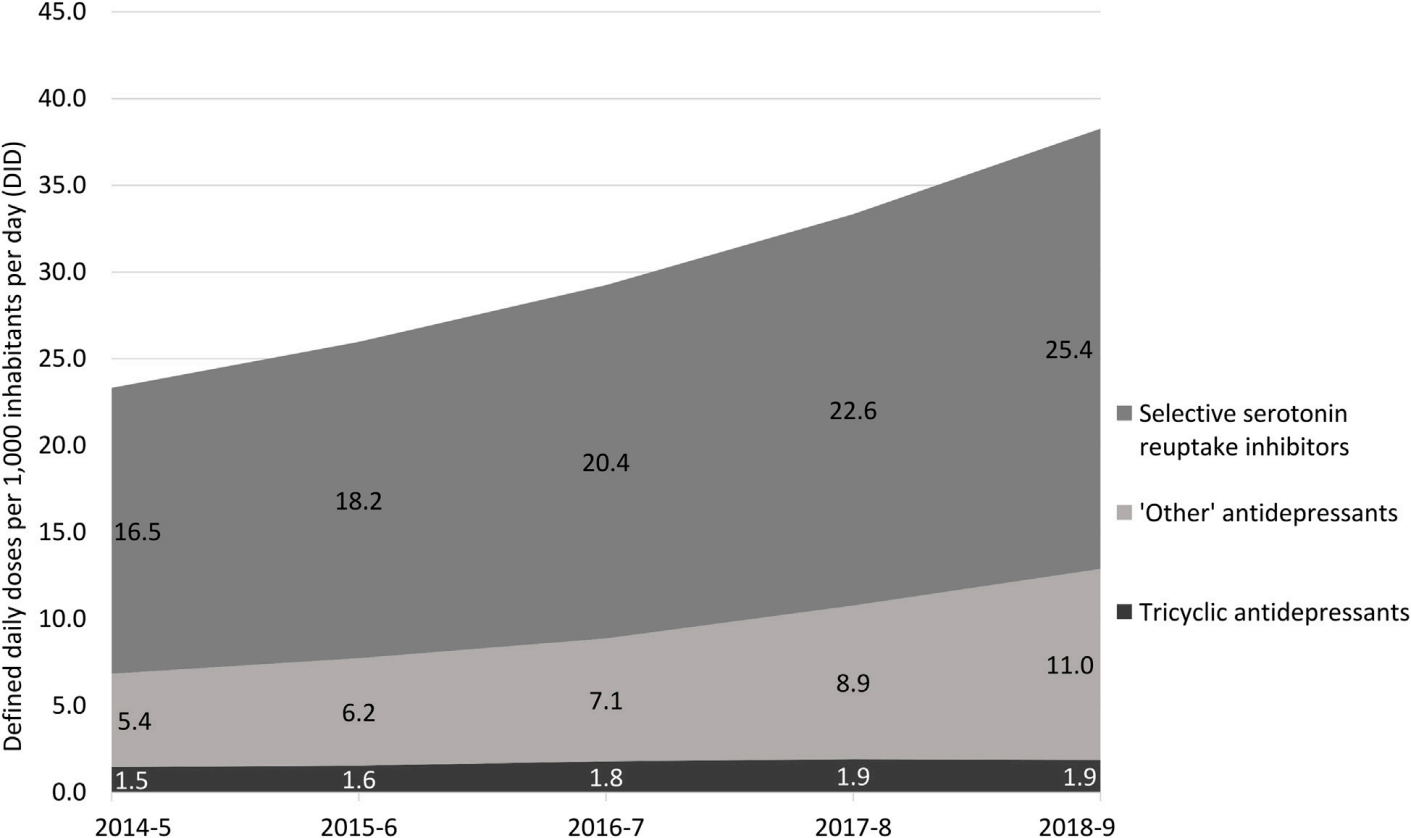
Sales of antidepressants in Brazil from November 2014 to October 2019 in defined daily doses per 1,000 inhabitants per day (DID) according to the main pharmacologic categories. Note: monoamine oxidase inhibitors were excluded of this analysis due to low DID (<0.1).

Total sales of antidepressants increased and sales of tricyclics remained almost unchanged, while those of selective serotonin reuptake inhibitors and ‘other’ antidepressants increased in the period. Monoamine oxidase inhibitors had DID <0.1 in the period (Figure 2).

Individually, the main sold antidepressants in terms of the total volume during the five-year period were escitalopram (+1.8 million DDDs), sertraline (+1.35 million DDDs), and fluoxetine (+1.09 million DDDs) (Table 1). The antidepressants that presented a higher sale growth rate were vortioxetine (growth rate: 336.2%), which was not sold in the first analyzed period (2014–5), followed by desvenlafaxine (growth rate: 285.2%) and duloxetine (growth rate: 121.4%) (Table 2). Maprotiline (-79.2%), moclobemide (-100%), and reboxetine (-100%) were the drugs with higher sale decrease (Table 2). The Figures 1, 2 illustrate variations of the antidepressant sales in the studied period and deserves attention to escitalopram, in both absolute and relative terms, whose sales had increased from 4.4 DIDs to 9.0 DIDs (growth rate: 104.1%, p = 0.029). Monoamine oxidase inhibitors experienced sale reduction (−9.5%).

## Discussion

The total sales of antidepressants had increased above two-thirds from November 2014 to October 2019 in Brazil. Selective serotonin reuptake inhibitors had higher sale volume, highlighted by escitalopram, and the ‘other’ antidepressant category had a higher growth rate in the period, mainly due to increase of new marketed drugs such as desvenlafaxine (three-fold increase) and duloxetine (two-fold increase). On the other hand, monoamine oxidase inhibitors experienced sale reduction, especially moclobemide.

It is important to consider some limitations of our results. The period of analysis of this study potentially was not enough to analyze associations among the sale trends and possible triggers factors (e.g., dates of launching a new antidepressant, introduction of first generic of each antidepressant, publication of depressive disorder guidelines, and inclusion of antidepressants in the national essential drug list, among other contextual factors) (Luo et al., 2020; Nguyen et al., 2020; Luo et al., 2021). The nationally aggregated data also prevented us from investigating associations among regional factors (e.g., the Human Development Index, per capita income, demographic characteristics, and unemployment index) and the antidepressant commerce profile. Furthermore, it was not possible to test associations among fluctuations of antidepressant sales and individual demographics of the patients or prescribers. The wide variety of uses for these drugs (e.g., depression disorders, anxiety, neuropathic pain, and others) and lack of prescription data prevented us from establishing a direct relationship between sale trends and the prevalence of depressive disorder treatment. Despite this, it is reasonable to assume the results as a proxy of use, especially by converting product packages to DDDs, a global standardized metric that allows trends in drug utilization to be assessed and comparisons among regions and populations be performed (Blix, 2016). The analyzed database provides data from hospitals, pharmacies, wholesalers, and drug companies, from private and public sectors, and represents a comprehensive view of the pharmaceutical market in Brazil.

Antidepressant sales increased in almost 70% in our analysis. Increase in the use of antidepressants from 2014 to 2019 was also observed in other countries, members of the Organization for Economic Co-operation and Development, but at a lower rate: Canada (39%), Chile (20%), Estonia (49%), Iceland (27%), Israel (28%), Latvia (60%), Portugal (35%), and Sweden (17%), among others (Organization for Economic Co-operation and Development, 2020). The use of antidepressants has been increasing in the last decades worldwide, suggesting that affective disorders are getting more common or more diagnosed or even drug companies could be increasing efforts to promote these drugs (Alduhishy, 2018). The increase of antidepressant use also may reflect improvements of mental health services and implementation of national policies that promote drug affordability, such as the generic drug policy. Other possible factors to be considered are difficulty to discontinue antidepressants, which may prolong the treatment period beyond the recommended by the best scientific evidence (Morrison, 2010; Davies and Read, 2019), and possible misuse (e.g., inappropriate indication, duration, or type of drug) of the psychopharmaceuticals (Devos et al., 2020).

Selective serotonin reuptake inhibitor sales increased in the period and represented about two-thirds of total antidepressant sales in Brazil. Escitalopram, licensed in 2002 in Brazil, doubled its sales from 2014 to 2019. A similar pattern was observed in Uruguay from 2010 to 2014, with escitalopram as the most used antidepressant (Domínguez et al., 2017). Selective serotonin reuptake inhibitors were also the most used antidepressants in Poland (Bliźniewska-Kowalska et al., 2020), Belgium (Devos et al., 2020), the Netherlands (Huijbregts et al., 2017), Iran (Soleymani et al., 2018), the United States (Luo et al., 2020), and China (Yu et al., 2020) in periods that ranged from 1995 to 2018.

Escitalopram was the most sold active substance in total volume in Brazil during the assessed period, followed by sertraline and fluoxetine. Most of the antidepressants had their respective generic introduced in Brazil before 2014, but desvenlafaxine and agomelatine had their generics introduced during the studied period, in 2016 and 2018, respectively. Moclobemide, tianeptine, maprotiline, reboxetine, milnacipran, and vortioxetine did not have a generic introduced in the country until October 2019. Sertraline and fluoxetine were also among the most frequently prescribed antidepressants from 1996 to 2015, in the United States, while citalopram and escitalopram were prescribed by an expressive large amount soon after their entry into the market, in 2000 and 2005, respectively (Luo et al., 2020). The risk of causing QT prolongation followed a prescription decrease for both escitalopram and citalopram in the United States (Luo et al., 2021) and in the United Kingdom (Godman et al., 2019). Concerns of cardiovascular safety apparently did not affect citalopram and escitalopram sales in Brazil. According to our finding, citalopram had stable sales, while escitalopram had doubled its sale volume in Brazil in the assessed period. In our study, replacement from citalopram to escitalopram was not possible to be observed because escitalopram was launched before 2014, but the shift was observed in the United States around 2005, after citalopram just lost its patent (Luo et al., 2021).

In 2005, the U.S. Food and Drug Administration published a warning for duloxetine due to liver side effects, but it seems not to have caused a prescription decrease in that country (Luo et al., 2021). In 2010, after the Swedish Dental and Pharmaceutical Benefits Agency (TLV) had limited the duloxetine prescription to refractory patients, a significant increase of venlafaxine utilization in Sweden was observed, which was not observed after generic venlafaxine introduction one year before in that country (Godman et al., 2013). In our study period, duloxetine was the antidepressant with the third highest sale growth rate and venlafaxine sales had not increased significantly, but denvenlafaxine had an expressive sale increase of 285%, probably associated to generic introduction, in 2016.

An apparent gradual replacement of the traditional antidepressants (tricyclics and monoamine oxidase inhibitors) by newer ones (selective serotonin reuptake inhibitors and ‘other’ antidepressants) can be outlined in Brazil. A cohort of employees from Brazilian universities (Alcantara et al., 2020) observed a decrease in tricyclic use from 60% in 1999 to 15% in 2012, while selective serotonin reuptake inhibitor use increased from 29% to 67%, and ‘other’ antidepressants increased from 5% to 13% in the same period. A similar result was observed in a cross-sectional study of antidepressant utilization in all Iranian relevant population from 2006 to 2013 (Soleymani et al., 2018).

To the best of our knowledge, this study is a pioneer effort in assessing antidepressant sales in recent years in Brazil. Previous Brazilian assessments of psychotropic drugs relied on data from the Unified Health System (Sistema Único de Saúde, SUS), available for the SUS’s specialized component of pharmaceutical care, whose components are newer or more expensive drugs and thus do not represent the therapeutic arsenal for the condition (Barbosa et al., 2018). Our assessment is useful for estimating levels of exposure of certain populations to the effects of these technologies, their benefits, and damages. Further studies could assess longer periods and include patient, prescriber data, and price data to improve the understanding of such important market in a public health view.

The total sales of antidepressants increased from November 2014 to October 2019 in Brazil. The higher sales of selective serotonin reuptake inhibitors and higher growth rate of ‘other’ antidepressants, with low sales of tricyclics and a decrease of monoamine oxidase inhibitors, suggest the replacement of older drugs by newer ones, following a worldwide trend. Further studies may clarify the reasons for the sale trends of these drugs in Brazil.

## Data Availability

The raw data supporting the conclusion of this article will be made available by the authors, without undue reservation.
